# The Role of Gender Role Attitudes and Immigrant Generation in Ethnic Minority Women’s Labor Force Participation in Britain

**DOI:** 10.1007/s11199-018-0922-8

**Published:** 2018-04-28

**Authors:** Senhu Wang

**Affiliations:** Department of Sociology, University of Cambridge, Free School Lane, Cambridge, CB2 3RQ UK

**Keywords:** Ethnic minorities, Gender role attitudes, Labor force participation, Labor market, Immigrant generation, Immigrants, Women

## Abstract

Although substantial research shows that in Britain some ethnic minority women have significantly lower labor force participation (LFP) rates than White British women, even after controlling for demographic characteristics and education levels, little is known about the reasons underlying the remaining ethnic differences. Using nationally representative data (2010–2011), I investigate the role of gender role attitudes in explaining the ethnic as well as generational differences in women’s LFP rates. The results show that after controlling for demographic characteristics and education levels, LFP rates of Pakistani and Bangladeshi women are significantly lower than that of White British women and about half of the ethnic gap can be explained by differences in gender role attitudes. Moreover, I show that the ethnic gap is less pronounced for second generation Pakistani, Bangladeshi, and Indian women whose LFP rates are significantly higher than those of their first generation counterparts. Importantly, the higher LFP rates of second generation South Asian women can be largely explained by their relatively less traditional gender role attitudes. Drawing on my results, public policies could provide appropriate childcare services and flexible work arrangements to alter traditional gender role attitudes, thereby improving minority women’s labor market opportunities.

**Electronic supplementary material:**

The online version of this article (10.1007/s11199-018-0922-8) contains supplementary material, which is available to authorized users.

In recent decades, persistent economic disadvantage of some British ethnic minority women has become an increasingly important concern of public policy. In Britain, it is well documented that South Asian women (defined as Indians, Pakistanis, and Bangladeshis) often have significantly lower levels of labor force participation (LFP) than White British women do (Dale and Ahmed 2010; Dale et al. 2008). In contrast, Black Caribbean and Black African women are found to have similar or higher levels of LFP compared to White British women (Dale et al. 2008; Peach 2005). The divergent women’s LFP rates among British ethnic groups are thought to lead to significant ethnic and gender inequalities in a wide arrange of socio-economic domains (Kan and Laurie 2016).

For example, it is argued that LFP could significantly improve not only women’s empowerment and increase opportunities in other domains such as education and health (Kan and Laurie 2016; Khoudja and Fleischmann 2017), but also their household decision-making power and children’s well-being (Majlesi 2016). For immigrant and minority women, LFP could also promote inter-ethnic interactions, thereby facilitating successful ethnic integration and reducing ethnic discrimination and prejudices (Heath et al. 2013). In contrast, low LFP rates of minority women could exacerbate their subordinated family position and social status, leading to durable gender inequalities in a variety of socio-economic domains (Dale and Ahmed 2010). Given the importance of minority women’s LFP, successive policies and legislations such as the recent Equality and Human Rights Commission in 2007 and Equality Act in 2010 have been implemented in order to tackle ethnic and gender discrimination and inequalities in the labor market (APPG 2017; Casey Review 2016; Zuccotti 2015).

The 2016 Casey Review into social integration in Britain suggests that differences in women’s LFP among British ethnic groups could be partly due to cultural differences in what are considered to be appropriate roles and behaviors for women (Aston et al. 2007; Heath et al. 2013). For example, research suggests that South Asian women (defined as Pakistanis, Bangladeshis, and Indians) often have traditional gender role attitudes characterized by a clear household division of labor between a male breadwinner and female homemaker, and they also have lower levels of LFP than other ethnic groups do (Aston et al. 2007; Dale and Ahmed 2010). By contrast, Black Caribbean and Black African women with more egalitarian gender role attitudes in terms of the household labor division tend to have similar or even higher levels of LFP than White British women do (Kan and Laurie 2016; Peach 2005). Although these studies imply that gender role attitudes could be an important reason underlying ethnic differences in women’s LFP, it remains unclear about the extent to which gender role attitudes could explain ethnic differences in women’s LFP after taking into account demographic characteristics and education levels. Therefore, my first objective is to extend previous research by exploring the role of gender role attitudes in explaining divergent LFP rates among ethnic groups of British women.

Although South Asian women, especially Pakistanis and Bangladeshis, generally have relatively low LFP rates, recent research shows significant generational changes within South Asian women, with the second generations having much higher LFP rates than the first generations (Dale and Ahmed 2010; Heath et al. 2013). Most previous research attributes the increased LFP of second generation South Asian women to their higher levels of human capital and different demographic characteristics (Heath et al. 2008; Platt 2007). However, a number of qualitative studies show that in recent decades some South Asian communities have witnessed a value shift from traditional to relatively more egalitarian gender role attitudes and an improvement in women’s status (Aston et al. 2007; Dale et al. 2002), which in turn could improve South Asian women’s LFP. Despite these important findings, it remains unclear (a) whether the findings from qualitative research can be generalized beyond the studied communities and applied to the population of South Asian women in Britain and (b) the extent to which changes in gender role attitudes can explain generational differences in South Asian women’s LFP rates after taking into account other confounding factors. Thus, my second objective is to explore the role of gender role attitudes in explaining generational differences in LFP within South Asian women.

## Ethnicity and Women’s Labor Force Participation

Over the last few decades, large-scale immigration flows have made Britain a multi-ethnic society where immigrants and ethnic minorities account for 14% of the total population in 2011 (Zuccotti 2015). The major and well-established ethnic minority groups in Britain are Indians, Pakistanis, Bangladeshis, Black Caribbeans (mainly from Jamaica and its surrounding Caribbean islands), and Black Africans (mainly from Nigeria and other sub-Saharan African countries) (Heath et al. 2013). The increasing population share of immigrant ethnic minorities has stimulated heated discussion about whether these minorities could have equal labor market opportunities to the native White British population. For example, previous research shows divergent women’s LFP rates among British ethnic groups, with the three South Asian groups of women having significantly lower LFP rates than White British women. In contrast, Black Caribbean and Black African women have similar or higher LFP rates than White British women do (Dale and Ahmed 2010; Dale et al. 2008). Given the adverse impacts of low LFP on women’s outcomes, understanding and explaining such ethnic differences becomes particularly crucial.

Currently, dominant explanations of women’s LFP often highlight the importance of human capital as well as demographic and family characteristics. For example, household specification theory argues that men and women tend to maximize their joint household utility by specializing in the labor market and domestic housework respectively (Becker 1991). This is mainly because women often have relatively lower payoff and potential earnings than their male partners (Becker 1991). Thus, human capital (e.g., education) that could improve women’s potential payoff in the labor market has been widely regarded as an important factor promoting women’s LFP. Moreover, women with younger dependent children are more likely to withdraw from the labor market than those with older children who can assist them with domestic housework (Kan and Laurie 2016; Read 2004). Similarly, women who live in large extended families are less likely to participate in the labor market because other family members could contribute to household income, reducing the need for female employment (Read 2004). Finally, household and partner’s economic status have been found to be positively associated with women’s LFP, although some other scholars claim the opposite (see Khoudja and Fleischmann 2017 for a review).

However, recent research shows that demographic characteristics and education levels cannot sufficiently explain ethnic differences in women’s LFP. For example, research from Britain shows that after controlling for demographic characteristics and education levels, some South Asian women, in particular Pakistani and Bangladeshi women, still have significantly lower LFP rates than their White British counterparts do (Dale and Ahmed 2010; Dale et al. 2008). For these minority groups, the unexplained ethnic differences net of demographic characteristics and education levels are termed as “ethnic penalties,” which are thought to be caused by multiple reasons such as poor language proficiency, a lack of bridging social networks, cultural norms, and discrimination (Heath and Cheung 2007). In the previous research, ethnic penalties are often regarded as an important indicator of “net” ethnic differences or inequalities in labor market outcomes (Heath and Cheung 2007). As a result, high levels of ethnic penalties are likely to exacerbate ethnic and gender inequalities in a wide arrange of socio-economic domains (Kan and Laurie 2016; Khoudja and Fleischmann 2017; Majlesi 2016).

## Ethnicity and Gender Role Attitudes

Recent research suggests that an important reason for ethnic penalties or differences in women’s LFP could be differences in gender role norms and attitudes concerning the appropriate social roles and responsibilities of men and women (Casey Review 2016). Because attitudes help shape behavior, gender role attitudes can have gender and ethnic inequalities in multiple life course domains, ranging from time use, educational enrolment, labor force, and occupational choices (Kan and Laurie 2016; Khoudja and Fleischmann 2017; Read 2004). In recent decades, many Western countries have witnessed a gender value shift as traditional attitudes emphasizing a gendered division of household labor between a male breadwinner and female homemaker have weakened in favor of a more egalitarian and individualist model that is characterized by higher women’s LFP and a more equal sharing of household care duties and domestic tasks (Berridge et al. 2009).

These macro-social trends may, however, be affected by the influx of immigrants from culturally distinct backgrounds. Previous research suggests that gender role attitudes do vary by ethnicity in Britain. For example, South Asian minorities, especially Pakistanis and Bangladeshis, are often found to exhibit more traditional gender role attitudes than White British do; by contrast, Black Caribbeans and Black Africans are found to have more egalitarian attitudes than White British do (Berthoud 2005; Dale and Ahmed 2010). It is often argued that these divergent gender role attitudes are jointly shaped by differences in the home country’s cultural traditions, migration histories, and levels of discrimination (Heath et al. 2013). In the following sections, I discuss how each of the three factors affects gender role attitudes for the five ethnic minority groups I study.

### Cultural Traditions

The divergent gender role attitudes of British ethnic minorities are significantly shaped by their different cultural traditions. Peach (2005) and Kan and Laurie (2016) suggest that both Black Caribbean and Black African women have more egalitarian gender role attitudes than White British women do and that these egalitarian attitudes are partly shaped by the matriarchal family traditions in Afro-Caribbean culture where oldest women are the head of the family. However, another stream of research shows that Black Caribbeans have even more egalitarian attitudes than Black Africans because their attitudes were also influenced by the inherited egalitarian and individualist gender ideology forged during historic experiences of West Indian slavery when husbands and wives were usually sent to different plantations and assigned similar workloads (Berthoud 2005). As a result, Black Caribbeans are thought to have the most egalitarian gender role attitudes in Britain (Berthoud 2005). This is not only exhibited in their egalitarian gender division of housework and care duties (Kan and Laurie 2016), but is also related to their low marriage rates (39% as compared with 60% of White British) and their high incidence of lone parenthood (50% as opposed to 20% of White British) (Berthoud 2005; Dale and Ahmed 2010; Platt 2010).

In contrast, South Asian women, especially Pakistanis and Bangladeshis, tend to have more traditional gender role attitudes and lower LFP rates than White British do. These more traditional views are often thought to be rooted in a more patriarchal home country culture, as well as adherence to relatively conservative strands of religions (Heath et al. 2013). Most Pakistanis and Bangladeshis, and a substantial proportion of Indians, are Muslims, whereas other Indians are Hindus and Sikhs. Research suggests that all three religious groupings have a relatively patriarchal and traditional system of gender relations (Heath et al. 2013). In these cultural and religious systems, men tend to dominate gender relations and act as the family breadwinner, whereas women have a relatively subordinated position in the family and take care of household tasks, child rearing, and care duties (Aston et al. 2007; Dale et al. 2002; Shaw 2000). Moreover, high in-group marriage rates within South Asian cultures, which often involve marriage to an immigrant partner from the home country (Casey Review 2016), could further help socialize and maintain traditional attitudes within families and minority communities.

### Migration Histories

Ethnic differences in gender role attitudes are also influenced by their different migration histories and experiences. Black Caribbeans are a relatively selective and well-established multigenerational immigrant group who initially came to Britain to fill post-war labor shortages during a period of steady economic growth in the 1950s and 1960s (Heath et al. 2013). Similarly, waves of Black Africans have been migrating to Britain for higher education since the 1950s, and they also have a long tradition of migration and are a well-established group (Heath et al. 2013). Thus, period effects and a longer tradition of migration and settlement in Britain as well as high education levels might combine to explain why Black Caribbean and Black African women now have a more favorable labor market position and more egalitarian gender role attitudes than other ethnic groups do.

In contrast, Pakistani and Bangladeshi migrants initially came to Britain during the 1960s to 1980s to fill jobs in declining industries such as textiles or unskilled construction in East London, the North-West, and parts of South-East England (Heath et al. 2013) An important feature of this immigration stream was that Pakistani and Bangladeshi men came first and settled down before their wives followed later as dependents whose immigration was permitted through family reunification policies (Dale and Ahmed 2010; Dale et al. 2008). Recent work shows that this means that Pakistani and Bangladeshi women continue to have lower levels of human capital, worse labor market attainments, and a weaker proficiency with English than both their male counterparts and women from other minority groups (Casey Review 2016). These factors are likely to perpetuate traditional gender role attitudes and low LFP rates.

Finally, Indians are a more heterogeneous minority group with diverse migration histories. The partition of India in 1947 displaced an initial flow of Indian migrants to Britain, followed shortly by another wave recruited as doctors during the 1950s and 1960s. More recent Indian migrants were former government officials and businessmen who were expelled from Uganda in the early 1970s (Dale and Ahmed 2010; Heath et al. 2013). These migration experiences suggest that Indians overall have a more favorable socio-economic position and higher levels of human capital than Pakistanis and Bangladeshis do. Thus, Indians are likely to hold less traditional gender role attitudes than Pakistanis and Bangladeshis, but still more traditional than White British.

### Discrimination

Finally, all ethnic minority women are likely to suffer discrimination from at least four aspects: sexism, immigrant minority status, colorism/racism, and religion (Dale et al. 2008; Heath et al. 2013). These factors could lead to ethnic disadvantages in workplace and hiring as well as discourage out-group interactions, potentially exacerbating their low levels of LFP and perpetuating traditional gender role attitudes and divisions of labor (Heath and Demireva 2014). However, Black Caribbeans’ and Black Africans’ egalitarian gender role attitudes and high LFP rates suggest that they are less affected by discrimination than South Asians who have highly traditional attitudes and low LFP rates. There are three reasons to explain these ethnic discrepancies. First, as we have seen, South Asian women have much lower social status, education levels, and worse migration backgrounds compared with Black Caribbean and Black African women. This could exacerbate gender and immigrant discrimination in the workplace and further impede their LFP. Second, Black Caribbeans and Black Africans are socially integrated into British society with high levels of inter-ethnic friendships and marriages with the host society’s members, whereas South Asians often have ethnically homogenous social networks and high in-group marriage rates (Heath et al. 2013). Previous research shows that a lack of social integration could reinforce racism and discrimination, preventing minorities from assimilating mainstream values and attitudes (Schlueter 2012). Third, because a large proportion of South Asians are Muslims, they are more vulnerable to discrimination and harassment than other minority groups due to recent Islamophobia in Europe (Heath and Demireva 2014). This could discourage travel and out-group interactions, reinforcing their minority identities and traditional gender role attitudes. These three factors, taken together, suggest that discrimination from the four domains could significantly reinforce the traditional attitudes of South Asian women and impede their LFP.

In sum, in multicultural Britain, South Asian women, especially Pakistanis and Bangladeshis, have more traditional gender role attitudes than White British women do, whereas Black Caribbean and Black African women have more egalitarian (or less traditional) attitudes than White British women do. These divergent gender role attitudes are likely to play an important role in explaining ethnic differences in women’s LFP in addition to demographic characteristics and human capital. However, it is important to note that the association between gender role attitudes and women’s LFP is often confounded by other factors such as age, partnership, presence of dependent children, and education levels. Thus, my first objective is to investigate the extent to which gender role attitudes explain ethnic differences in women’s LFP while taking into account demographic characteristics and education levels. Based on previous research, I hypothesize that, net of demographic characteristics and education levels, ethnic differences in women’s LFP are significantly explained by gender role attitudes (Hypothesis 1).

## Generational Differences in Women’s LFP and Gender Role Attitudes

Although South Asian women, especially Pakistanis and Bangladeshis, generally have relatively low LFP rates, recent research shows significant generational changes within the South Asian women, with the second generation having much higher levels of LFP than the first generation (Dale and Ahmed 2010; Dale et al. 2008). Most previous research tends to attribute such generational differences in women’s LFP to the higher levels of human capital of second generation minority women. For example, it is argued that second generation minority women often have fewer language difficulties as well as higher and more recognizable educational qualifications than their first generation counterparts do (Heath et al. 2013). These factors not only could increase their potential labor market payoff, but also could help them to avoid some potential employer discrimination (Heath et al. 2008). Moreover, research shows that many second generation South Asian women tend to marry and have children at an older age, as well as prefer fewer children than the first generation (Dale et al. 2002). These demographic differences could be another important factor that could explain the higher LFP rates among the second generation minorities.

Although human capital and demographic differences matter for the generational differences in women’s LFP, there is no known research exploring whether changes in gender role attitudes can explain generational differences in women’s LFP. In fact, there are a number of qualitative studies, which show that there is a value shift over generations within these communities such that the younger generations who were grew up in Britain having increasingly less traditional gender role attitudes than their parental-generation counterparts (Aston et al. 2007; Dale et al. 2002). In qualitative research on female family and gender roles in some South Asian communities, Aston et al. (2007) find that the value shift is partly due to the fact that the second generation women received education in Britain and might have more exposure to the gender role attitudes of the majority group, which is relatively less traditional (also see Heath et al. 2013). More importantly, Aston et al. show that compared with their parents’ generations, the second generation minority women who were born in Britain are subject to different expectations from families and communities, and they are often given more freedom and flexibility in terms of their education and career choices.

Moreover, many male elders who are often powerful leaders in Pakistani and Bangladeshi communities become more tolerant about female employment and place increasing emphasis on their daughters’ education regardless of their own educational backgrounds (Aston et al. 2007; Dale et al. 2002). They think that education and employment could give their daughters more opportunities, making them more confident, independent, and sociable: “they would listen to their daughters and give them freedom because they would want them to be happy with the choices they made for themselves” (Aston et al. 2007, p. 101). Thus, as a result of both receiving socialization in the host country and being subject to a different set of family and community expectations, the second generation South Asian women are very likely to assimilate the relatively less traditional gender role attitudes of White British and have higher LFP rates than their first generation counterparts (Dale et al. 2002). Despite these important findings, it is worth exploring whether the results from the qualitative research can be generalized to the population of South Asian women in Britain and remain valid after adjusting for other confounding factors. Thus, my second hypothesis states that, net of demographic characteristics and education levels, differences in LFP between first and second generation South Asian women are significantly explained by differences in gender role attitudes (Hypothesis 2).

## Method

### Data and Sample

The data used in my study come from the second wave (2010–2011) of the United Kingdom Household Longitudinal Study (UKHLS). I use this wave of the survey because the adults’ self-completed questionnaire asked respondents about gender role attitudes. UKHLS comprises a stratified and clustered General Population (GP) sample of around 40,000 households as well as an Ethnic Minority Boost (EMB) oversample, which was designed to yield at least 1000 respondents for five major ethnic minority groups: Indian, Pakistani, Bangladeshi, Black Caribbean, and Black African (Knies 2016). The interview response rates in this wave were 61% and 46% for the GP and EMB samples respectively (Knies 2016). Among the interviewed respondents, 89% of the GP and 72% of the EMB sample completed the adult self-completion questionnaire (Lynn et al. 2012). Thus, cross-sectional weights provided by UKHLS were used to adjust for unequal non-response and selection probabilities.

To construct the analytical sample, I first excluded male respondents from the sample because my study focuses on women’s LFP. Moreover, full-time students and those who are older than 65 (the retirement age) or younger than 18 were excluded because these groups are potentially inactive in the labor force and thus not involved in making trade-offs between paid work and domestic labor. Minority groups other than those sampled in EMBS were discarded due to very small sample sizes. After dropping a very small number of observations with missing values (around 3%), the final estimation sample contained 17,614 women of whom 15,635 were White British; 441, Pakistani; 226, Bangladeshi; 563 Indian; 382 Black African; and 367 Black Caribbean, al aged 18–65 living in Britain.

### Measures

#### Ethnicity

As the UK Office of National Statistics (ONS 2003, p. 9) argues, “membership of an ethnic group is something that is subjectively meaningful to the person concerned, and this is the principal basis for ethnic categorisation in the United Kingdom.” Thus, in my study ethnicity is measured by self-identified ethnic classification. Specifically, the questionnaire asked respondents to select from a list of 18 ethnic categories the ethnic group to which they think they belong (for more details, see understandingsociety.ac.uk). These ethnic categories are established by using both national (e.g., Indian) and a supra-national approach (e.g., African; Caribbean) in order to achieve a balance among ancestry, culture, nationality, language, religion, and so on (ONS 2003). Because this ethnic classification is widely used in Census and other large-scale survey data in Britain, my study uses this classification for easy comparability with previous research.

In my study, White British are defined as the respondents who identify themselves as “White British/English/Scottish/Northern Irish.” Similarly, the five minority groups (i.e., Pakistani, Bangladeshi, Indian, Black African, and Black Caribbean) are identified using the same approach. The five minority groups are selected not only because they are the largest minority groups in Britain, but also because UKHLS has an over-sample for these groups. I follow previous research (e.g., Heath et al. 2008) to further distinguish the five minority groups between first (born overseas) and second or later generation (born in or arrived in the UK before the age of 7). The average age of arrival for the five minority groups is 23.62 (*SD* = 11.22, range = 1–65). Due to data limitation (there are no appropriate variables, e.g., ethnicity and birthplace of respondents’ parents and grandparents), I was unable to further distinguish between second and later generations. Nevertheless, because these second generation groups were socialized in Britain, it is appropriate to assume that they share relatively similar characteristics (Heath et al. 2013).

#### Labor Force Participation (LFP)

The dependent variable is LFP rate or economic activity rate, which refers to the proportion of the population (excluding full-time students) who is either employed or unemployed, but has looked for a job during the last 2 weeks or could start to work within 2 weeks. The variable is coded as a binary variable (0, No; 1, Yes).

#### Gender Role Attitudes

Gender role attitudes are derived from responses to three self-completed survey questions: “A pre-school child is likely to suffer if his or her mother works”; “All in all, family life suffers when the woman has a full time job’; and “A husband’s job is to earn money; a wife’s job is to look after the home and family.” (For more details, see understandingsociety.ac.uk.) Respondents were asked to rate each item on a 5-point scale ranging from 1 (*strongly disagree*) to 5 (*strongly agree*). Because the three items are ordinal variables, standard factor analysis that is based on Pearson’s correlations and assumes that variables are continuous could lead to bias (Holgado–Tello et al. 2010). Thus, the polychoric factor analysis using polychoric correlations was employed to extract one factor from these three variables, which explained 71.7% of the total variance with eigenvalue >1 and α = .83. Further analysis shows that the Cronbach’s alpha of gender role attitudes is consistent across ethnic groups (ranges from.81 to.85). To facilitate interpretation, the factor score was standardized to range from 0 to 10, where a higher score refers to more traditional gender role attitudes.

#### Control Variables

A range of individual variables include age, age squared, and partnership that is coded as a binary variable to indicate whether individuals have a partner. A binary variable captures whether respondents have pre-school children under 5-years-old. I also control for education, which is coded into three categories: university/college degree or above; high school (A-level and GCSE) or lower; and no qualification.

### Analytic Strategy

Because the dependent variable (LFP) is binary, I use logistic regression models. Because I aim to compare coefficients across logistic regression models, it is important take into account the scaling problem, which is likely to bias the cross-model comparison (Mood 2010). I follow Mood (2010) to use Average Marginal Effects (AME) that could effectively solve this problem. Moreover, AME are more intuitive to interpret than odds ratios because they show the change of expected probability of the dependent variable with one unit increase in the independent variable. I begin by comparing the raw differences in LFP rates between White British and ethnic minority women. I then add age and generation cohort, demographic characteristics, and education levels stepwise into the models in order to explore net ethnic differences or ethnic penalties. Next, the models further include gender role attitudes to explore the extent to which gender role attitudes could explain such net differences in women’s LFP (to test Hypothesis 1). Finally, I repeat the prior analysis but restrict the sample to ethnic minorities in order to explore the extent to which gender role attitudes could explain differences in LFP between first and second generation South Asian women (to test Hypothesis 2).

## Results

### Descriptive Statistics

Table 1 presents descriptive statistics disaggregated by ethnicity and minority generation. The *F* or Chi-squared statistics (depending on whether the variable is continuous or categorical) show that all variables significantly differ across ethnic and generational groups (*p* < .001). Specifically, I find that whereas first generation South Asian women have lower LFP rates than White British women do, first generation Black Caribbean and Black African women have similar LFP rates to White British women. Moreover, all second generation minorities have higher LFP rates than their first generations. In particular, second generation Indian, Black Caribbean, and Black African women have even higher LFP rates than White British women do, whereas second generation Pakistani and Bangladeshi women still have lower LFP rates than White British women. Moreover, both first and second generation South Asian women have more traditional gender role attitudes than White British women. In contrast, both first and second generation Black Caribbean and Black African women have more egalitarian attitudes than their White British counterparts. Importantly, all second generation minorities have less traditional attitudes than their first generation counterparts.

**Table 1:** Descriptive statistics by ethnic and generational group

	White British	Pakistani		Bangladeshi		Indian		Black Caribbean		Black African		*F/*χ^*2*^ *p*-value
		1st gen.	2nd gen.	1st gen.	2nd gen.	1st gen.	2nd gen.	1st gen.	2nd gen.	1st gen.	2nd gen.	
LFP (%)												
Yes Gender role attitudes (*M*)	71.87 4.74	25.24 6.93	51.49 5.65	26.19 6.63	67.32 5.38	58.89 5.87	78.45 4.92	73.02 4.12	80.49 3.94	7.18 4..64	80.00 4.36	<.001 <.001
(*SD*)	(2.06)	(1.98)	(1.89)	(2.21)	(2.17)	(2.13)	(1.99)	(2.27)	(1.91)	(1.98)	(1.98)	
Age (*M*)	43.88	39.27	32.05	37.91	3.29	42.34	35.20	43.94	41.29	39.21	34.62	<.001
(*SD*)	(12.88)	(1.24)	(1.85)	(9.76)	(9.66)	(1.79)	(9.29)	(9.76)	(8.76)	(9.72)	(1.00)	
Partnership (%)												
Yes	69.03	84.29	64.26	88.10	6.40	86.01	65.16	43.65	28.57	52.71	34.00	<.001
Preschool age children (%)												
Yes	16.78	41.43	43.29	41.27	4.00	27.70	27.27	15.20	2.66	39.46	28.00	<.001
Education (%)												
Degree or above	35.82	21.43	39.15	17.46	35.64	52.19	51.58	33.33	51.02	5.60	7.00	
High school or lower	53.55	39.52	54.47	49.21	59.41	32.36	45.25	52.38	46.53	34.34	28.00	
No qualification	1.64	39.05	6.38	33.33	4.95	15.45	3.17	14.29	2.45	15.06	2.00	<.001
*n*	15,635	210	231	126	100	343	220	125	242	332	50	

Although the descriptive findings hint that gender role attitudes are an important reason to explain ethnic and generational differences in women’s LFP, Table 1 also shows clear ethnic and generational differences in various compositional factors. In terms of demographic characteristics, all ethnic minorities except Black Caribbeans are younger than White British women, and second generations as a whole are generally younger than the first generations. Moreover, whereas South Asian women, especially first generation Pakistanis and Bangladeshis, are more likely to have a partner than White British, Black Caribbeans and Black Africans have lower partnership rates than White British women. All ethnic minorities except Black Caribbeans are more likely to have preschool age children than White British do. Finally, all ethnic minorities except first generation Pakistanis and Bangladeshis have higher education levels than White British, and there is a clear generational improvement in education levels for all ethnic minorities.

### Regression Analysis

Table 2 reports average marginal effects (AME) of a series of logistic regression models comparing LFP rates between White British and ethnic minority women. Model 1 compares the raw differences and shows that the LFP rates of Pakistani, Bangladeshi, and Indian women are 26, 26, and 7% points lower than that of White British women (*p* < .001), whereas Black Caribbean women have 9% points higher LFP rate than that of White British (*p* < .001), and Black Africans have a similar LFP rate to White British (*p* = .361). With age and generation cohort entered, Model 2 shows that for the general population, there is a curvilinear relationship between age and women’s LFP with younger and older women having lower LFP than middle-aged women (*p* < .001). Model 2 also shows that, overall, second generation minority women have significantly higher LFP rates than the first generation (*p* < .001).

**Table 2:** Average marginal effects of logistic regression models predicting women’s labor force participation

Predictors	Model 1	Model 2	Model 3	Model 4	Model 5
Ethnicity (ref = White British)					
Pakistani	−.26***	−.25***	−.20***	−.16***	−.08**
(*SE*)	(.03)	(.03)	(.03)	(.03)	(.03)
*p*	.000	.000	.000	.000	.006
Bangladeshi	−.26***	−.24***	−.19***	−.16***	−.09*
(*SE*)	(.05)	(.04)	(.04)	(.04)	(.04)
*p*	.000	.000	.000	.000	.012
Indian	−.09***	−.02	−.01	−.02	.00
(*SE*)	(.02)	(.02)	(.02)	(.02)	(.02)
*p*	.000	.309	.481	.293	.826
Black Caribbean	.09***	.09**	.08**	.07*	.05
(*SE*)	(.02)	(.02)	(.02)	(.03)	(.03)
*p*	.000	.001	.002	.014	.102
Black African	−.03	−.03	.01	.01	.03
(*SE*)	(.03)	(.03)	(.03)	(.03)	(.03)
*p*	.361	.294	.591	.736	.296
Age		−.10***	−.13***	−.11***	−.11***
(*SE*)		(.00)	(.00)	(.00)	(.00)
*p*		.000	.000	.000	.000
Age^2^		−.05***	−.04***	−.04***	−.04***
(*SE*)		(.00)	(.00)	(.00)	(.00)
*p*		.000	.000	.000	.000
Generation (ref = 1st gen.)		.07***	.05***	.05***	.04**
(*SE*)		(.02)	(.02)	(.02)	(.02)
*p*		.000	.007	.004	.035
Partnership (ref = No)			.01	−.00	−.00
(*SE*)			(.01)	(.01)	(.01)
*p*			.370	.648	.899
Preschool age children (ref = No)			−.29***	−.29***	−.27***
(*SE*)			(.01)	(.01)	(.01)
*p*			.000	.000	.000
Education (ref = Degree or above)					
High school or lower				−.08***	−.06***
(*SE*)				(.01)	(.01)
*p*				.000	.000
No qualification				−.23***	−.21***
(*SE*)				(.01)	(.01)
*p*				.000	.000
Gender role attitudes					−.03***
(*SE*)					(.00)
*p*					.000
Observations *R* ^2^	17,614 .02	17,614 .07	17,614 .16	17,614 .19	17,614 .23

Average Marginal Effects (AMEs) show the change of expected probability of the dependent variable with one unit increase in the independent variable

* *p* < .05. ** *p* < .01. *** *p* < .001

Moreover, in Model 2 the regression coefficient becomes nonsignificant for Indian women, but remains similar for other minority groups. This suggests that the lower LFP rate of Indian women relative to White British women is mainly due to the age and cohort effects. Model 3 further includes partnership and presence of children, and it shows that women having preschool age children have significantly lower LFP than those who do not (*p* < .001). With these variables entered, ethnic differences in LFP decrease by 5% points for Pakistanis and Bangladeshis, as well as 1% point for Black Caribbeans, suggesting that ethnic differences in women’s LFP are partly explained by differences in individual demographic characteristics.

Next, Model 4 further includes education and shows that women with a degree tend to have significantly higher LFP rates than those with lower education levels (*p* < .001). With education entered into the model, ethnic differences in LFP further decrease by 4 and 3% points for Pakistanis and Bangladeshis, and 1% point for Black Caribbeans. This suggests that ethnic differences in women’s LFP are partly due to differences in education levels. So far, the ethnic differences net of important demographic characteristics and education are −16% for Pakistanis and Bangladeshis (*p* < .001), and 7% for Black Caribbeans (*p* = .014). In other words, both Pakistani and Bangladeshi women suffer significant ethnic penalties in LFP relative to White British women, whereas Black Caribbean women enjoy an ethnic advantage in LFP compared to White British women.

Model 5 includes gender role attitudes and shows that traditional gender role attitudes are significantly associated with lower women’s LFP rates (*p* < .001). Importantly, including gender role attitudes reduces around half of the ethnic differences for Pakistani and Bangladeshi women, although their LFP rates are still 8% (*p* = .006) and 9% (*p* = .012) points lower than that of White British women. Moreover, with gender role attitudes entered, the ethnic difference between Black Caribbean and White British women becomes nonsignificant. This pattern suggests that an important reason for Caribbean women’s higher LFP rate relative to White British is their relatively egalitarian gender role attitudes. Finally, the *R*^2^ increases from 19% to 23%, again highlighting the importance of gender role attitudes for women’s LFP.

In addition, I conducted two robustness checks. First, I included interaction terms between ethnicity and gender role attitudes while controlling for demographic characteristics and education (see Table 1s in the online supplement). None of the interaction effects were significant, suggesting that the association between gender role attitudes and women’s LFP did not vary across different ethnic groups. Second, I re-ran all analyses while restricting the sample only to respondents with a partner (see Table 2s in the online supplement). Generally, I find that the results remain similar to those reported here, but ethnic differences are more pronounced for Pakistani and Bangladeshi women in the restricted models. This difference suggests that having a partner is more likely to impede women’s LFP for both groups than for White British.

Table 3 repeats Models 4–5 in Table 2 for the three South Asian groups, and it explores whether gender role attitudes can explain generational differences in women’s LFP rates within the minority population. Models A1, B1 and C1 show that after controlling for demographic characteristics and education, all three South Asian groups experience a significant improvement in women’s LFP over generations. Specifically, the generational difference in LFP rate is 15% for Pakistani women (*p* = .001), 18% for Bangladeshi women (*p* = .006), and 16% for Indian women (*p* = .001). In terms of demographic characteristics and education, I find a curvilinear relationship between age and women’s LFP for Indians (*p* = .028), but not for Pakistanis and Bangladeshis. Moreover, I find that having a partner is significantly and negatively associated with lower women’s LFP rates for Pakistanis (*p* < .001) and Bangladeshis (*p* = .019), but not for Indians. Finally, the presence of preschool age children and lower education levels are significantly and negatively associated with lower women’s LFP rates for all minority groups.

**Table 3:** Average marginal effects of logistic regression models predicting labor force participation of first and second generation south asian women

Predictors	Pakistani		Bangladeshi		Indian	
	Model A1	Model A2	Model B1	Model B2	Model C1	Model C2
Generation (ref = 1st gen.)	.15**	.07	.18**	.06	.16**	.09*
(*SE*)	(.05)	(.04)	(.06)	(.04)	(.05)	(.04)
*p*	.001	.119	.006	.220	.001	.022
Age	−.05	−.04	−.06	−.06	−.09***	−.09***
(*SE*)	(.04)	(.04)	(.05)	(.05)	(.02)	(.02)
*p*	.236	.252	.227	.232	.000	.000
Age^2^	.01	.01	.01	.01	−.03*	−.03*
(*SE*)	(.02)	(.02)	(.03)	(.03)	(.01)	(.01)
*p*	.393	.413	.728	.794	.028	.021
Partnership (ref = No)	−.39***	−.37***	−.21*	−.21*	.02	.02
(*SE*)	(.06)	(.06)	(.09)	(.09)	(.06)	(.05)
*p*	.000	.000	.019	.015	.705	.643
Preschool age children (ref = No)	−.20***	−.19***	−.27**	−.26**	−.28***	−.26***
(*SE*)	(.05)	(.05)	(.09)	(.09)	(.05)	(.05)
*p*	.000	.000	.002	.003	.000	.000
Education (ref = Degree or above)						
High school or lower	−.13*	−.12*	−.18*	−.17*	−.09*	−.08*
(*SE*)	(.06)	(.06)	(.09)	(.08)	(.04)	(.04)
*p*	.020	.029	.042	.036	.019	.045
No qualification	−.36***	−.35***	−.42***	−.41***	−.21**	−.18**
(*SE*)	(.09)	(.09)	(.12)	(.12)	(.07)	(.07)
*p*	.000	.000	.000	.001	.004	.008
Gender role attitudes		−.02*		−.02**		−.02**
(*SE*)		(.01)		(.01)		(.01)
*p*		.024		.001		.001
Observations	441	441	226	226	563	563
*R* ^2^	.20	.23	.20	.24	.18	.21

Average Marginal Effects (AMEs) show the change of expected probability of the dependent variable with one unit increase in the independent variable

* *p* < .05. ** *p* < .01. *** *p* < .001

Models A2, B2 and C3 include gender role attitudes and show that traditional gender role attitudes are significantly associated with lower women’s LFP rates for all minority groups (*p* < .001). With gender role attitudes entered, Models A2 and B2 show that generational differences for Pakistani and Bangladeshi women become nonsignificant. Moreover, Model C2 shows that, for Indian women, including gender role attitudes reduces generational difference by 7% points, although the difference remains significant (*p* = .022). I re-ran the analyses in Table 3 based on respondents with a partner (see Table 3s in the online supplement). The results are similar to those reported here, again highlighting the importance of gender role attitudes in patterning generational differences in women’s LFP within South Asian minorities. Finally, after including gender role attitudes the *R*^2^ increases by 3–4% for all minority groups, highlighting the importance of gender role attitudes for South Asian minority women’s LFP.

## Discussion

Using nationally representative survey data, I aimed to explore the role of gender role attitudes in shaping labor force participation (LPF) rates of British women from different ethnic groups. Overall, I find that, net of demographic characteristics and education levels, gender role attitudes play an important role in explaining differences in women’s LFP rates across different ethnic groups and within South Asian minorities. Specifically, I obtained this finding by testing two hypotheses.

My first hypothesis posited that gender role attitudes could significantly explain differences in women’s LFP rates between different British ethnic groups. This hypothesis is strongly supported. I first find that, net of demographic characteristics and education, Pakistani and Bangladeshi women have significantly lower LFP rates than White British women do and suffer significant ethnic penalties, whereas Black Caribbean women have significantly higher LFP rates than White British women do. These results are generally consistent with previous research suggesting diverse labor market experiences across different British ethnic groups (Dale and Ahmed 2010; Dale et al. 2008). More importantly, I show that about half of the ethnic penalties of Pakistanis and Bangladeshis can be explained by relatively traditional gender role attitudes. The unexplained ethnic penalties may be caused by other disadvantages related to ethnicity and migration such as poor language proficiency, unfamiliarity with the local labor market, a lack of bridging social networks, and so on (Heath et al. 2013). By contrast, the higher LFP rate of Black Caribbean relative to White British women is primarily due to the former’s more egalitarian gender role attitudes. Overall, these findings suggest that gender role attitudes play an important role in explaining divergent LFP rates among women from diverse British ethnic groups.

Moreover, recent research shows significant generational changes within the South Asian women, with the second generations having much higher levels of LFP than the first generations (Dale and Ahmed 2010; Dale et al. 2008). Although previous research tends to attribute the higher LFP rates of South Asian women to their higher levels of human capital and different demographic characteristics, there is no known research exploring the role of gender role attitudes in explaining such generational differences. Thus, my second hypothesis posited that the less traditional gender role attitudes of the second generation South Asian women is an important factor to explain their higher LFP rates relative to the first generations.

Overall, the second hypothesis is strongly supported. I first show that the second generations of the three South Asian groups not only have significantly higher LFP rates than the first generations net of demographic features and education levels, but also have less traditional gender role attitudes. This result suggests that South Asian women are integrating into the host society in terms of both LFP and gender role attitudes. Importantly, my results show that gender role attitudes can explain a large proportion of generational differences in women’s LFP for the three South Asian groups. Especially for Pakistanis and Bangladeshis with gender role attitudes entered, the net generational differences become nonsignificant. Overall, these results are consistent with previous qualitative studies (Aston et al. 2007; Dale et al. 2002), and they highlight that cultural assimilation in gender role attitudes is particularly important for the improvement of Pakistani and Bangladeshi women’s LFP. However, the generational difference remains significant for Indian women even after taking into account gender role attitudes. This suggests that other unobserved characteristics of the second generation Indian women may also contribute to their higher LFP rate such as better language proficiency and residence in less deprived areas.

### Practice Implications

Given the important role of gender role attitudes in patterning ethnic and generational differences in women’s LFP rates, my research holds significant implications for policymaking in terms of reducing ethnic and gender inequalities in the labor market. First, because the traditional gender role attitudes of minority women can result from disadvantaged migration backgrounds and discrimination by the host society, policymakers could provide skill and language training to help women overcome initial obstacles in the host labor market and implement regulations to tackle implicit discrimination against women and immigrant minorities in hiring and in the workplace. Moreover, the relatively liberal and less regulated labor market in Britain means that there are few flexible work arrangements, which could reinforce traditional gender role attitudes and labor division of some minority women (Gallie 2007). Thus, ensuring flexible and part-time employment opportunities could help them juggle work-and-life balance and increase their LFP rates. Finally, provision of appropriate childcare services could also help minority women with dependent children return to work. However, instead of providing homogeneous services to different population groups, it is important to take into account the distinctive religious beliefs of some minority women and provide culturally sensitive childcare, which is regarded by minority mothers as appropriate (Aston et al. 2007).

### Limitations and Future Research Directions

Despite these significant policy implications, there are several limitations of my study, which help focus future research. First, the current ethnic classifications used in my study may conceal some important within-group diversity in terms of religious affiliations and migration histories (for example). This could lead to inaccurate generalizations across ethnic groups. Future research using other datasets with a larger sample of ethnic minorities could further disaggregate each minority group and profitably explore more nuanced associations between their cultural values and LFP. Second, the measure of gender role attitudes used in my study primarily focuses on whether women should do paid work and may not reflect the total sum of gender relations and gender role division. Further research may use more comprehensive measures of gender role attitudes to examine their links to ethnicity and labor market outcomes. In addition, comparing gender differences in gender role attitudes and LFP, as well as exploring how these relationships vary across ethnic groups, is a promising topic and could shed light on the on-going discussion about persistent gender and ethnic inequalities in Britain. Finally, due to data limitations, my study used cross-sectional data and therefore is unable to explore causal and dynamic effects of gender role attitudes on women’s LFP. Doing so will require extending my work by using long periods of panel data to model long-term implications of gender role attitudes for ethnic minorities’ socio-economic trajectories.

### Conclusions

In Britain, the persistently low labor force participation rates of ethnic minority women have become an increasingly important concern of public policy, and they are thought to exacerbate gender and ethnic inequalities in a wide arrange of domains. Whereas previous studies emphasized the importance of demographic characteristics and human capital for women’s LFP, I show that traditional gender role attitudes are a crucial factor impeding women’s LFP, especially for Pakistani and Bangladeshi women. This result makes an important contribution to previous research by providing a new perspective to understand women’s LFP in a multi-ethnic society. However, it is important to note that traditional gender role attitudes are not the fixed and essentialized features of particular ethnic groups. Instead, I find that gender role attitudes are less traditional among second generation South Asian women, and these less traditional attitudes largely explain their higher LFP rates. This finding highlights the need to regard ethnicity and gender role attitudes as a fluid and changing characteristic, and it implies that the relationships among ethnicity, gender role attitudes, and women’s LFP are more nuanced than previously thought. Thus, a deeper exploration of such nuanced relationships could be a promising research area and provide important implications for future public policy.

## Electronic supplementary material

ESM 1 (DOCX 32 kb)
