# Supplementary material for: The Role of Gender Role Attitudes and Immigrant Generation in Ethnic Minority Women’s Labor Force Participation in Britain
Source: Sex Roles. 2018 Apr 28;80(3):234–45. doi: 10.1007/s11199-018-0922-8 (PMC6404549; doi:10.1007/s11199-018-0922-8)
Supplement: Supplementary file 1 — (DOCX 32 kb) [file 11199_2018_922_MOESM1_ESM.docx]

Online supplement for Wang, S. (2018). The role of gender role attitudes and immigrant generation in ethnic minority women’s labor force participation in Britain. *Sex Roles*. Senhu Wang, University of Cambridge**.** Email: [sw768@cam.ac.uk](mailto:sw768@cam.ac.uk)

| **Table 1s**  **Interactions between ethnicity and gender role attitudes** | | | |
| --- | --- | --- | --- |
| Predictors | Coef. | S.E. | p-values |
| **Ethnicity (ref = White British)** |  |  |  |
| Pakistani | -.90* | (.43) | .037 |
| Bangladeshi | -.73 | (.57) | .202 |
| Indian | .01 | (.32) | .967 |
| Black Caribbean | .05 | (.37) | .896 |
| Black African | .36 | (.48) | .449 |
| **Gender role attitudes (GRA)** | -.19*** | (.01) | .000 |
| **Ethnicity * GRA** |  |  |  |
| Pakistani * GRA | **.00** | **(.07)** | **.966** |
| Bangladeshi * GRA | **-.02** | **(.10)** | **.866** |
| Indian * GRA | **.00** | **(.05)** | **.947** |
| Black Caribbean * GRA | **.09** | **(.08)** | **.233** |
| Black African * GRA | **-.03** | **(.08)** | **.719** |
| **Age** | -.77*** | (.03) | .000 |
| **Age^2^** | -.29*** | (.01) | .000 |
| **Generation (ref = 1^st^ gen.)** | .27* | (.13) | .034 |
| **Partnership (ref = No)** | -.01 | (.06) | .891 |
| **Preschool age children (ref = No)** | -1.79*** | (.07) | .000 |
| **Education (ref = Degree or above)** |  |  |  |
| High school or lower | -.43*** | (.05) | .000 |
| No qualification | -1.34*** | (.08) | .000 |
| **Constant** | 2.66*** | (.15) | .000 |
| **Observations** | 41,734 |  |  |
| **R^2^** | .23 |  |  |
| * *p* < .05. ** *p* < .01. *** *p* < .001. | | | |

| **Table 2s**  **Average marginal effects of logistic regression models predicting LFP of respondents**  **with a partner** | | | | | |
| --- | --- | --- | --- | --- | --- |
| Predictors | Model 1 | Model 2 | Model 3 | Model 4 | Model 5 |
| **Ethnicity (ref = White British)** |  |  |  |  |  |
| Pakistani | -.30*** | -.30*** | -.25*** | -.22*** | -.17*** |
|  | (.03) | (.03) | (.03) | (.03) | (.03) |
|  | .000 | .000 | .000 | .000 | .000 |
| Bangladeshi | -.31*** | -.30*** | -.24*** | -.21*** | -.18*** |
|  | (.05) | (.04) | (.04) | (.04) | (.04) |
|  | .000 | .000 | .000 | .000 | .000 |
| Indian | -.05 | -.06* | -.03 | -.03 | -.01 |
|  | (.03) | (.03) | (.03) | (.03) | (.03) |
|  | .104 | .036 | .215 | .192 | .818 |
| Black Caribbean | .11*** | .11** | .10*** | .09** | .07 |
|  | (.04) | (.04) | (.03) | (.03) | (.04) |
|  | .000 | .004 | .000 | .001 | .102 |
| Black African | -.05 | -.08* | -.02 | -.02 | -.01 |
|  | (.04) | (.04) | (.04) | (.04) | (.04) |
|  | .250 | .049 | .614 | .607 | .865 |
| **Age** |  | -.09*** | -.12*** | -.11*** | -.10*** |
|  |  | (.00) | (.00) | (.00) | (.00) |
|  |  | .000 | .000 | .000 | .000 |
| **Age^2^** |  | -.07*** | -.05*** | -.05*** | -.05*** |
|  |  | (.00) | (.00) | (.00) | (.00) |
|  |  | .000 | .000 | .000 | .000 |
| **Generation (ref = 1^st^ gen.)** |  | .04 | .03 | .04 | .02 |
|  |  | (.02) | (.02) | (.02) | (.02) |
|  |  | .062 | .137 | .083 | .330 |
| **Preschool age children (ref = No)** |  |  | -.26*** | -.27*** | -.25*** |
|  |  |  | (.01) | (.01) | (.01) |
|  |  |  | .000 | .000 | .000 |
| **Education (ref = Degree or above)** |  |  |  |  |  |
| High school or lower |  |  |  | -.07*** | -.05*** |
|  |  |  |  | (.01) | (.01) |
|  |  |  |  | .000 | .000 |
| No qualification |  |  |  | -.21*** | -.18*** |
|  |  |  |  | (.02) | (.02) |
|  |  |  |  | .000 | .000 |
| **Gender role attitudes** |  |  |  |  | -.03*** |
|  |  |  |  |  | (.00) |
|  |  |  |  |  | .000 |
| **Observations** | 12,111 | 12,111 | 12,111 | 12,111 | 12,111 |
| **R^2^** | .02 | .11 | .18 | .21 | .25 |
| *Note*. Standard errors are reported in parentheses; *p*-values are reported below standard errors.  * *p* < .05. ** *p* < .01. *** *p* < .001. | | | | | |

| **Table 3s**    **Average marginal effects of logistic regression models predicting LFP of first and second generation South Asian women with a partner** | | | | | | |
| --- | --- | --- | --- | --- | --- | --- |
|  | Pakistani | | Bangladeshi | | Indian | |
| Predictors | Model A1 | Model A2 | Model B1 | Model B2 | Model C1 | Model C2 |
| **Generation (ref = 1^st^ gen.)** | **.16**** | **.06** | **.20**** | **.09** | **.17**** | **.14**** |
|  | **(.05)** | **(.05)** | **(.07)** | **(.12)** | **(.05)** | **(.05)** |
|  | **.001** | **.176** | **.005** | **.436** | **.001** | **.007** |
| **Age** | -.08 | -.07 | -.06 | -.06 | -.09*** | -.09*** |
|  | (.05) | (.05) | (.07) | (.07) | (.02) | (.02) |
|  | .108 | .119 | .424 | .393 | .000 | .000 |
| **Age^2^** | .00 | .01 | -.01 | -.02 | -.06*** | -.05** |
|  | (.02) | (.03) | (.04) | (.04) | (.02) | (.02) |
|  | .847 | .838 | .884 | .700 | .000 | .001 |
| **Preschool age children (ref = No)** | -.22** | -.22** | -.31** | -.30** | -.22*** | -.21*** |
|  | (.07) | (.07) | (.09) | (.09) | (.05) | (.05) |
|  | .001 | .002 | .001 | .001 | .000 | .000 |
| **Education (ref = Degree or above)** |  |  |  |  |  |  |
| High school or lower | -.13 | -.12 | -.18 | -.16 | -.08 | -.06 |
|  | (.08) | (.08) | (.13) | (.11) | (.05) | (.05) |
|  | .113 | .144 | .152 | .137 | .113 | .183 |
| No qualification | -.32** | -.32** | -.49** | -.49*** | -.17* | -.16* |
|  | (.11) | (.12) | (.14) | (.14) | (.08) | (.08) |
|  | .003 | .007 | .001 | .000 | .038 | .048 |
| **Gender role attitudes** |  | -.04** |  | -.03** |  | -.02* |
|  |  | (.01) |  | (.01) |  | (.01) |
|  |  | .009 |  | .001 |  | .013 |
| **Observations** | 328 | 328 | 172 | 172 | 439 | 439 |
| **R^2^** | .21 | .24 | .20 | .24 | .19 | .21 |
| *Note*. Standard errors are reported in parentheses; *p*-values are reported below standard errors.  * *p* < .05. ** *p* < .01. *** *p* < .001. | | | | | | |
